# Endogenous sex steroid hormones and colorectal cancer risk: a systematic review and meta-analysis

**DOI:** 10.1007/s12672-021-00402-z

**Published:** 2021-03-15

**Authors:** Emmanouil Bouras, Christopher Papandreou, Ioanna Tzoulaki, Konstantinos K. Tsilidis

**Affiliations:** 1 Department of Hygiene and Epidemiology, University of Ioannina Medical School, Ioannina, Greece; 2 Institut D′Investigació Sanitària Pere Virgili (IISPV), Reus, Spain; 3 Department of Epidemiology and Biostatistics, School of Public Health, Imperial College London, St Mary’s Campus, London, W2 1PG UK

**Keywords:** Estradiol, Testosterone, Sex-hormone binding globulin, Colorectal cancer, Systematic review, Meta-analysis

## Abstract

Preclinical data suggest that endogenous sex steroid hormones may be implicated in colorectal cancer (CRC) development, however, findings from epidemiological studies are conflicting. The aim of this systematic review and meta-analysis was to investigate the associations between endogenous concentrations of sex hormones and CRC risk. PubMed and Scopus were searched until June 2020 for prospective studies evaluating the association between pre-diagnostic plasma/serum concentrations of estradiol, testosterone and sex-hormone binding globulin (SHBG) and CRC risk. Summary relative risks (RRs) and 95% confidence intervals (CIs) were estimated using the inverse-variance weighted random-effects model based on the DerSimonian-Laird estimator. Eight studies were included in the meta-analysis after evaluating 3,859 non-duplicate records. Four of the eight studies had a nested case–control design, one study was a case-cohort and the rest three studies were cohort studies, and they included on average 295 cases (range:48–732) and 2,105 controls. No associations were found for endogenous sex steroid hormones in men or post-menopausal women with CRC risk, with evidence for substantial heterogeneity observed among women. Findings from this meta-analysis do not support presence of associations between pre-diagnostic concentrations of testosterone, estradiol and SHBG with incident CRC risk in men and post-menopausal women.

**Supplementary Information:**

The online version contains supplementary material available at 10.1007/s12672-021-00402-z.

## Introduction

Colorectal cancer (CRC) is the third most frequent cancer in males and second in females worldwide, with almost one million new incident cases in 2018 [1]. Sex differences in CRC development have been suggested [2, 3] and in support of this notion are the findings from several epidemiological studies [4–8] showing a higher incidence in men, with age-standardised incidence rates of 23.4 per 100,000 compared to 16.2 in females, worldwide [1, 9]. Furthermore, a protective role of exogenous hormones, such as oral contraceptives and postmenopausal hormone therapy, is observed for CRC risk [10–13]. However, the role of endogenous concentrations of sex hormones, such as estradiol, testosterone and sex-hormone binding globulin (SHBG), in CRC risk is unclear. Preclinical data suggest a potential role of estradiol and testosterone in the aetiology of CRC [14, 15]. Estrogen through its receptors have been shown to activate diverse intracellular pathways, including protein kinase C (PKC), intracellular Ca2+, cytosolic cAMP, nitric oxide and MAPK, that are implicated in cancer [16]. Experimental evidence also suggest that testosterone is implicated in colon cancer cell apoptosis, mediated through the activation of specific membrane-binding sites and the subsequent regulation of the PI3K/Akt pathway, apoptotic proteins of Bcl-2 family, Bad, and the reorganization of actin cytoskeleton through small GTPase signalling, including CDC42, RAC1 and RhoA/B [17]. A significant positive association between endogenous estradiol concentrations and CRC incidence was found among postmenopausal women in the Women's Health Initiative Observational Study (WHI-OS) [18]. However, two case–control studies, one nested within the New York University Women's Health Study (NYUWHS) [19] and another nested within 4 US cohort studies [2] reported no significant association between estradiol concentrations and CRC risk. Contradictory findings also exist in relation to circulating testosterone and CRC risk. Higher concentrations of testosterone have been associated with a decreased risk of CRC in men [2] and increased risk in postmenopausal women [20]. Other prospective studies examining this association showed null findings [21–24]. Similarly, conflicting results emerged on other components of the endogenous sex hormone axis, including SHBG, in two prospective studies (WHI-OS, 4 US studies) reporting no significant findings [2, 19],whereas a significant positive association was reported in the follow-up of the Women's Health Initiative Clinical Trial (WHI-CT) [25].

Summarizing the available evidence on the role of endogenous sex steroid hormones on CRC risk could assist in understanding better the hormonal perturbations associated with CRC development. Therefore, we conducted a systematic review and meta-analysis for the association of circulating endogenous concentrations of estradiol, testosterone and SHBG with CRC risk combining results from prospective epidemiological investigations conducted in men and women.

## Methods

### Literature search and data extraction

We searched for eligible published studies in MEDLINE-PubMed and Elsevier’s Scopus databases up to 17 June, 2020 using the search algorithm described in Additional file 1: Table S1. All studies obtained from the literature searches were uploaded in the web-based application of rayyan, which was used to manage the study selection process [26]. We considered only prospective epidemiological studies assessing the association between pre-diagnostic concentrations of endogenous sex steroid hormones, namely plasma or serum estradiol, testosterone and SHBG, and risk of developing CRC. We excluded non-human and in-vitro studies, narrative or systematic reviews, case-series or case-reports, cross-sectional studies, studies on CRC survival, studies conducted in children, duplicate studies, studies that did not provide enough information for calculating measures of association, and studies in other than the English language. When there were more than one studies from the same cohort, we selected the publication with the longer follow-up [21].

**Table 1:** Characteristics of the 8 prospective studies included in the meta-analysis examining the associations between endogenous sex hormones and the risk of colorectal cancer risk

Study	Country	Study design	Length of follow-up (years)^a^	Population	Cases	Controls	Age (years)^a^	BMI (kg/m^2)^a^	Exposure(s) of interest	Method of assessment	Adjustments
NYUWHS (Clendenen, T. V., 2009)	USA	Nested case–control	From 1985 to 2003	Post-menopausal women	148	293	Cases: 60.4; Controls: 60.4	Case: 25.6; Control: 24.7	Estradiol; SHBG	Estradiol; RIA following organic extraction and celite chromatography (LLOQ = 2 pg/mL); SHBG: Two-site immunometric chemiluminescent assay on an IMMULITE 2000 instrument (LLOQ = 2 nmol/L)	Age at enrolment, date of blood donation, BMI
Copenhagen City Heart Study (Orsted, D. D., 2014)	Denmark	Cohort	22	Men; Women^**b**^	Men: 185; Women: 84	Men: 4243; Women: 4217	Men: 58.0; Women: 57.0	Men: 25.6; Women: 24.0	Testosterone	Testosterone: Immunochemically using the ADVIA (reportable range of the assay 0.35–260 nmol/L)	Smoking status, cumulative smoking, BMI, alcohol consumption, level of education, and level of income. In women also adjusted for parity, menopausal status, oral contraceptive use, and hormone replacement therapy
WHI-CT (Murphy, N., 2015)	UK	Nested case–control	From 1993–98 to 2008	Post-menopausal women	401	802	Case: 66.0; Control: 66.0	Case: 28.5; Control: 28.4	Estradiol; SHBG	Estradiol: RIA following organic extraction and celite chromatography (LLOQ = 2 pg/mL); SHBG: RIA following organic extraction and celite chromatography (LLOQ = 10 pg/mL)	WC, alcohol consumption, history of CRC, physical activity, smoking status, and NSAID use
Busselton Health Study (Chan, Y. X., 2018)	Australia	Cohort	20	Men	48	1526	Overall: 51.1	Overall: 26.7	Testosterone; Estradiol; SHBG	Testosterone: single LC–MS/MS (CV of 8.6% at 5.3 nmol/L and 7.9% at 26.9 nmol/L; Estradiol: Single LC–MS/MS (CV of 14.5% at 73 pmol/L and 9.9% at 279 pmol/L.); SHBG: Two-site immunometric chemiluminescent assay on an IMMULITE 2000 instrument (CV of 3.4% at 39.4 nmol/L)	Age, marital status, occupation, smoking, alcohol consumption, leisure time physical activity, BMI and diabetes
HIMS (Chan, Y. X., 2017)	Australia	Cohort	9	Men	137	3436	Case: 77.9; Control: 76.9	Case: 26.4; Control: 26.5	Testosterone; Estradiol; SHBG	Testosterone: single LC–MS/MS run (CV of < 6% for T levels of > 0.4 nmol/l); Estradiol: single LC–MS/MS run (CV of < 8% for E2 levels of > 25 pmol/l); SHBG: chemiluminescent immunoassays (CV < 7%)	Age, BMI, smoking status, physical activity, duration of smoking, smoking exposure, alcohol, diabetes, HDL, TG, and history of cancer
JPHC (Mori, N., 2019)	Japan	Nested case–control	12	Post-menopausal women	185	361	Case: 60.0; Control: 59.8	Case: 23.8 Control: 23.5	Testosterone; Estradiol; SHBG	Testosterone: electrochemiluminescence (LLOQ < 0.226 ng/ml); Estradiol: electrochemiluminescence (LLOQ < 0.039 ng/ml); SHBG: chemiluminescence enzyme immunoassay;	BMI, smoking status, alcohol consumption, physical activity
NHS, WHS, HPFS, PHSII (Lin, J. H., 2013)	USA	Nested case–control	From (NHS:1989–90, WHS:1992, HPFS:1993–94, PHSII: 1986) to 2008	Men; Post-menopausal women	Men: 439; Women: 293	Men: 719; Women: 437	Case: 67.2; Control: 67.7	Case: 26.2; Control: 25.5	Testosterone; Estradiol; SHBG	Testosterone: competitive electrochemiluminescence immunoassay; Estradiol: turbulent flow liquid chromatography tandem mass spectrometry; SHBG: competitive electrochemiluminescence immunoassay; Mean intra-assay CVs 4%–7% in men and 3%–7% in women	Age, fasting status, hour at blood draw, smoking, alcohol intake, history of cancer, physical activity, history of polyps, and screening examination, BMI and C-peptide
WHI-OS (Gunter, M. J., 2008)	USA	Case-Cohort	6 (subcohort)	Post-menopausal women	438	809	Case: 65.9 Subcohort: 62.8	Case: 27.56; Subcohort: 27.07	Estradiol	Estradiol: Immunodiagnostic Assay	Age, smoking, ethnicity, family history of CRC, history of colonoscopy, physical activity, use of NSAIDs, and alcohol consumption

^**a**^Data are presented as mean or median values

^**b**^At baseline approximately 52% of women were post-menopausal and we expect that the majority of women would have been be post-menopausal at the time of cancer diagnosis, though this information was not reported in the paper

*NYUWHS* New York University Women’s Health Study, *WHI* Women’s Health Initiative, *CT* Clinical trial, *OS* Observational study, *CV* coefficient of variation, *HIMS* Health in Men Study, *JPHC* Japan Public Health Center-based Prospective Study, *NHS* Nurses’ Health Study, *WHS* Women’s Health Study, *HPFS* Health Professional Follow-up Study, *PHSII* Physicians’ Health Study II, *SHBG* Sex hormone binding globulin, *BMI*: Body mass index, *LLOQ* lower limit of quantitation, *WC* Waist-circumference, *CRC* Colorectal cancer, *HDL* High-density lipoprotein, *TG* Triglycerides, *NSAID* Nonsteroidal anti-inflammatory drugs, *RIA* Radioimmunoassay, *LC* liquid chromatography, *MS* mass spectrometry, *ng* Nano-grammar, *mL* milliliter, *nmol* nanomole, *L* Litre, *pmol* picomol

Screening of eligible studies and data extraction were performed independently by two investigators (EB and CP), and data was re-checked for consistency by a third author (KT). We initially removed duplicate publications from the electronic databases, and screened the titles and abstracts for eligibility in a blinded manner using rayyan. After the primary screening, the full texts of eligible studies were retrieved, and the inclusion and exclusion criteria were assessed. The “Newcastle–Ottawa Scale” [27] was used to assess the quality of the included studies. The assessment was based on the following criteria: *1*) study selection (maximum 4 points); *2*) adequacy of the exposure or outcome for nested case–control and cohort studies, respectively (maximum 3 points); and *3*) comparability of study groups (maximum 2 points). The studies were categorized as either high quality or low quality. The maximum score was 9, and a score of ≥ 6 indicated high methodological quality.

The data extracted from each eligible study included the following: first authors’ name and year of publication, country, study design, duration of follow-up, population characteristics such as age and sex, number of cases and controls, exposure and method of assessment, association estimates per level of exposure and adjustment factors. In case of multivariable adjusted statistical models, we retained the most adjusted model.

### Statistical analyses

Our primary analysis was a dose–response meta-analysis model considering continuous association estimates by sex. In studies, where association estimates per exposure were presented as three or more categories, generalized least-square regression was used to estimate the linear trends and express the association as per unit increase, using the method proposed by Greenland & Longnecker [28]. Specifically, we used the median concentrations of sex steroid hormones per tertile or quartile to represent the respective doses of the hormones. We used conversion factors (CF) based on the International System of Units (SI) to consider the doses under common units for each exposure (CF for estradiol: 1 pg/mL = 3.67 pmol/L;CF for testosterone: 1 ng/dL = 34.67 pmol/L).

The inverse-variance weighted random-effects DerSimonian-Laird meta-analysis [29, 30] was applied to estimate the pooled estimate for each of the associations of interest. Summary relative risks (RRs) and 95% CIs were calculated. A two-tailed *P* < 0.05 was considered statistically significant. The presence of heterogeneity was assessed using Cochran's Q test and the degree of heterogeneity was evaluated using the I^2^ metric. I^2^ values ≥ 50% indicated substantial heterogeneity [31]. Prediction intervals that provide an estimate of the interval within which a new study’s estimate would fall were also estimated as proposed by Higgins [32].

As a secondary analysis, we also meta-analysed the extreme quartiles of the endogenous sex hormone concentrations. All analyses were performed in R v.4.0.2 [33].

## Results

### Study selection

**Fig. 1:**
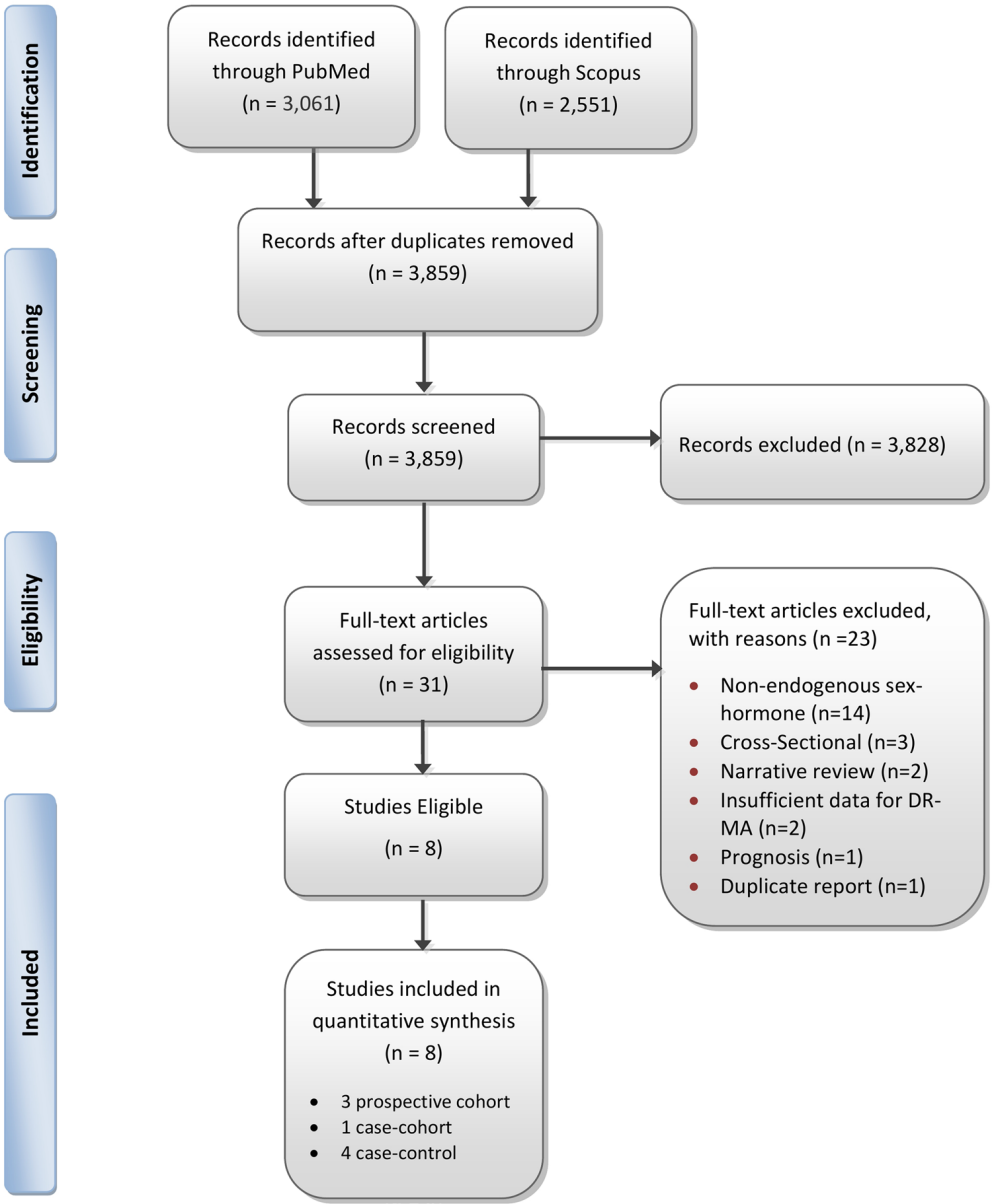
Flow diagram of studies assessed for eligibility per screening stage

The initial search yielded 3,859 non-duplicate records and after further screening, eight studies were deemed eligible for inclusion in the meta-analysis, namely three prospective cohorts [22–24], one case-cohort study [18] and four nested case–control studies [2, 19, 20, 25] (Fig. 1).

**Fig. 2:**
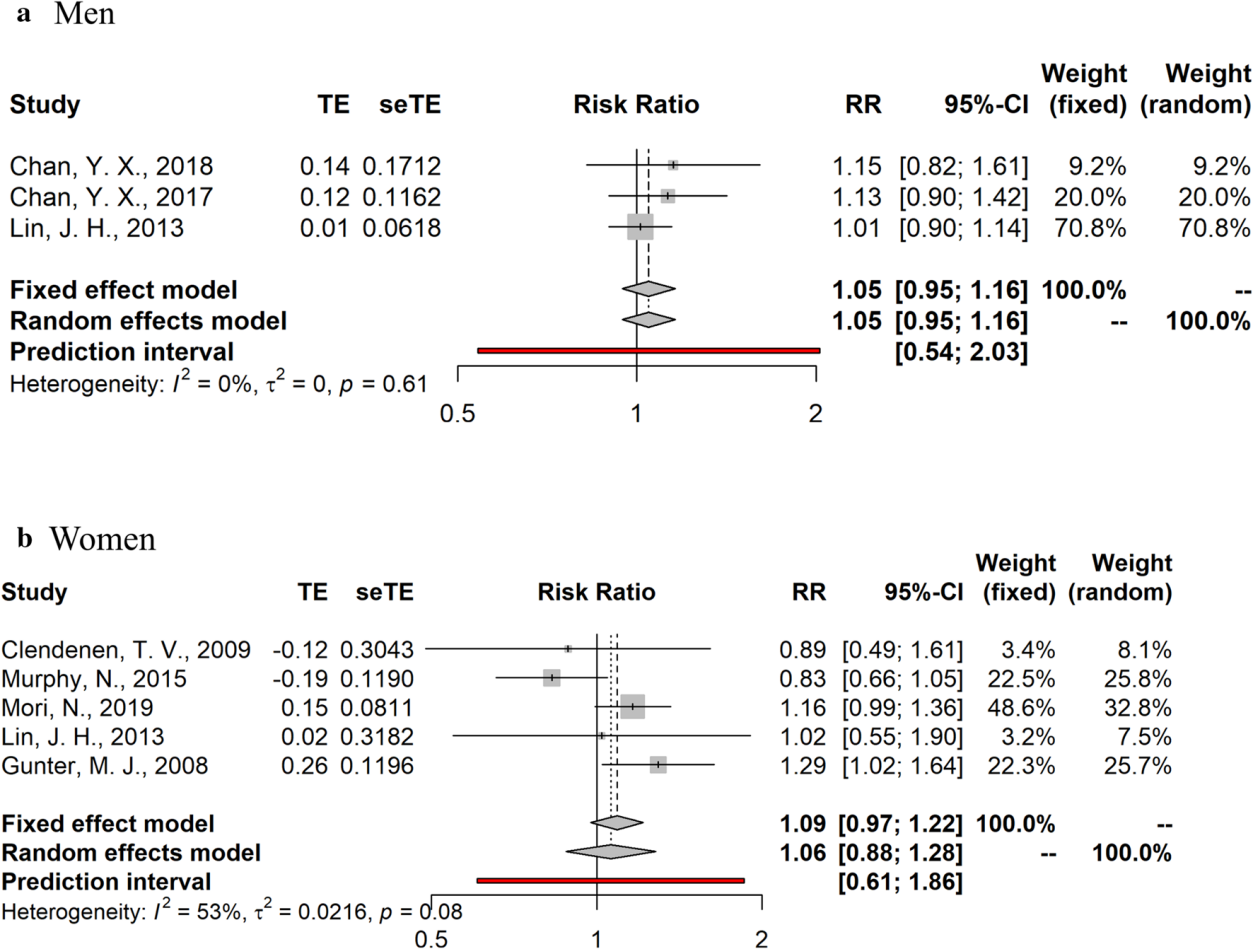
Summary relative risks and 95% confidence intervals of prospective studies for the association between ***estradiol*** and colorectal cancer risk in men and post-menopausal women. *CI* confidence interval, *RR* relative risk

**Fig. 3:**
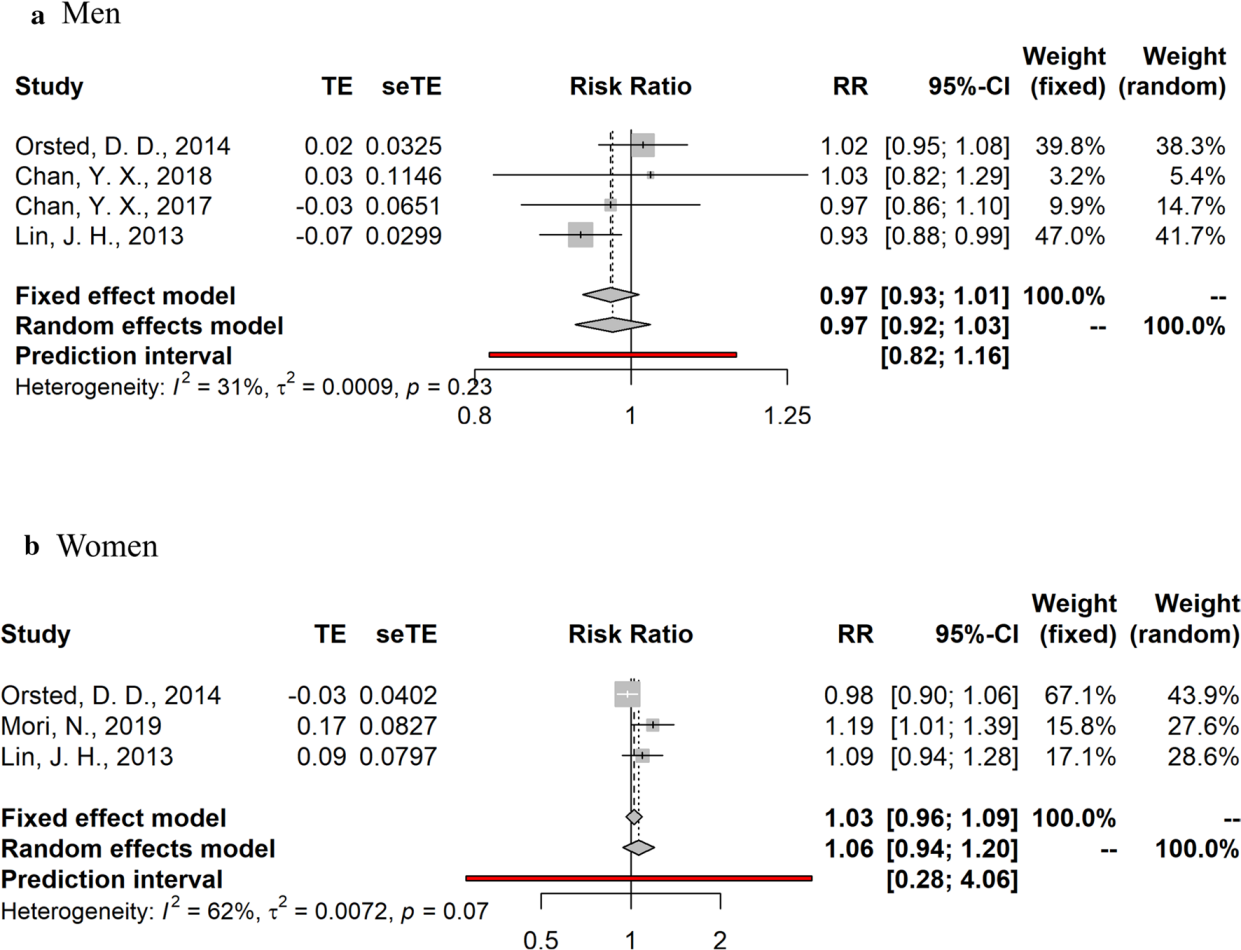
Summary relative risks and 95% confidence intervals of prospective studies for the association between ***testosterone*** and colorectal cancer risk in men and post-menopausal women. *CI* confidence interval, *RR* relative risk

**Fig. 4:**
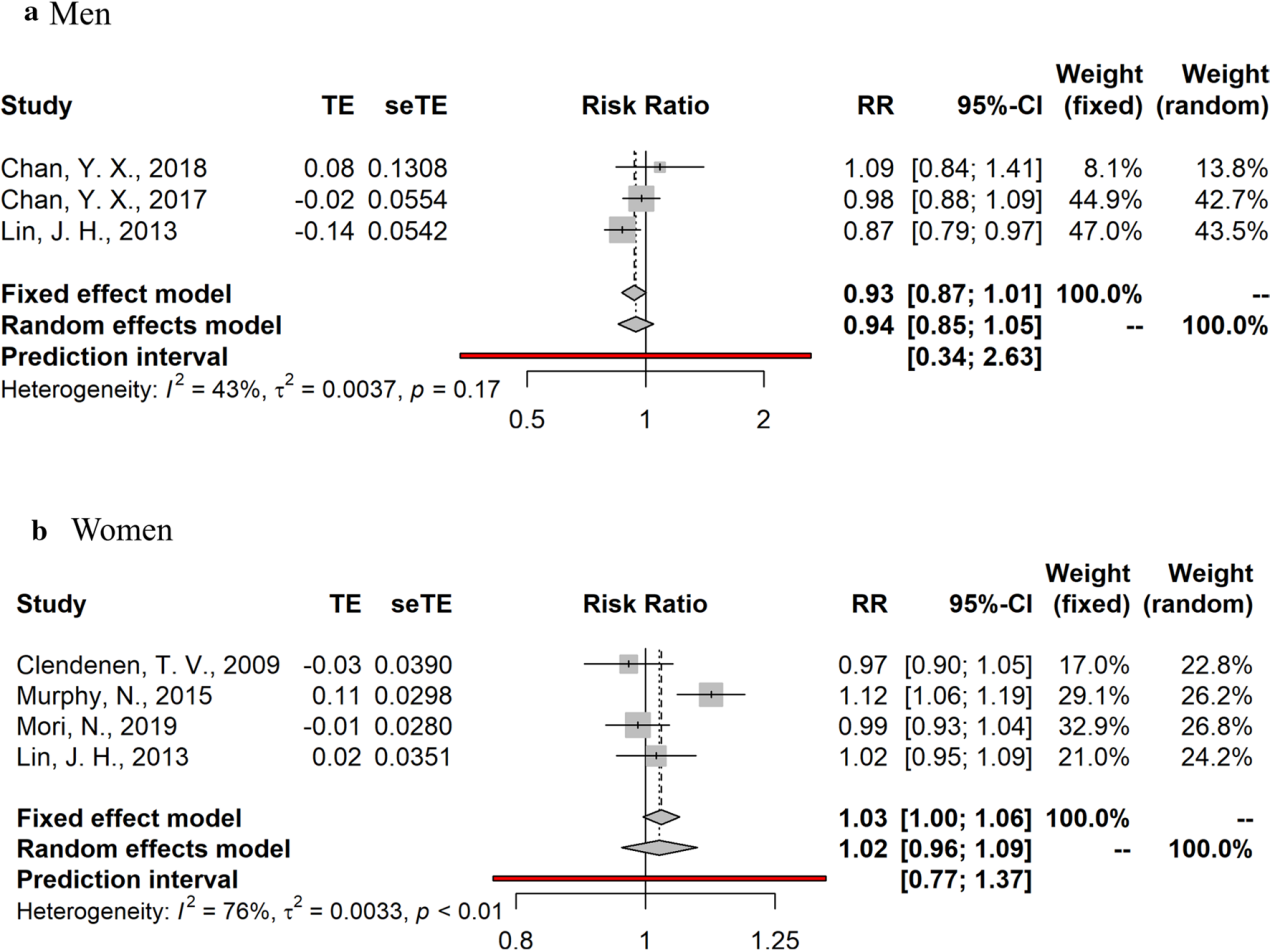
Summary relative risks and 95% confidence intervals of prospective studies for the association between sex-hormone binding globulin (***SHBG***) and colorectal cancer risk in men and post-menopausal women. *CI* confidence interval, *RR* relative risk

### Study characteristics

Table 1 shows the main characteristics of the selected studies. Three of the studies were from the USA, two from Australia, and one each from the UK, Denmark and Japan. The number of cases in the single studies ranged from 48 to 732. In total, the cohort studies included 13,876 participants with 454 cases, the case-cohort study included 438 cases and the nested case–control studies included 1,466 cases and 2,612 controls (Table 1). All of the included studies were of high quality with a mean of 8.5 points in the Newcastle–Ottawa scale (range: 7–9) (Additional file 1: Tables S2, S3).

### Evidence synthesis

Summary random- and fixed-effects RRs and 95% CIs, and heterogeneity statistics by sex are shown in Figs. 2, 3, 4. Due to the substantial heterogeneity among studies examining the relationship between endogenous sex hormones and CRC in women, we report results derived from the random-effects meta-analyses. No significant associations were observed in the dose–response meta-analyses for estradiol (RR per 10 pg/ml = 1.05, 95% CI: 0.95–1.16, I^2^ = 0% in men; RR per 10 pg/ml = 1.06, 95%CI: 0.88–1.28, I^2^ = 53% in post-menopausal women) and testosterone (RR per 100 ng/dL = 0.97, 95%CI: 0.92–1.03, I^2^ = 31% in men; RR per 10 ng/dL = 1.06; 95%CI: 0.94–1.20, I^2^ = 62% in post-menopausal women). SHBG was also not significantly associated with CRC risk (RR per 10 nmol/L = 0.94, 95%CI: 0.85–1.05, I^2^ = 43% in men; RR per 10 nmol/L = 1.02, 95%CI: 0.96–1.09, I^2^ = 76% in post-menopausal women).

In the comparisons of the top vs. bottom quartile of sex hormones concentrations, the meta-analyses showed a significant positive association between testosterone and CRC in post-menopausal women but only two studies were included (RR = 1.7, 95% CI: 1.11–2.6; I^2^ = 0%), while no significant associations were observed for estradiol or SHBG (Additional file 1: Figure S1).

## Discussion

Findings from this systematic review and meta-analysis summarized for the first time the association of pre-diagnostic concentrations of sex steroid hormones in relation to CRC risk. Eight prospective studies were meta-analyzed, but the summary findings did not support an association of estradiol, testosterone and SHBG with risk of CRC in men and post-menopausal women.

The discovery of different estrogen receptors (ER) have revealed a significant role in tissue types other than the female reproductive tract, including the gastrointestinal system [34]. There have been several in vitro studies reporting that estradiol may have tumorigenic effects on colorectal cells [35–37]. The mitogenic activities of estrogens are mediated through their intracellular receptors, ERα and ERβ [16]. Studies in male mice also suggest that testosterone may play a role in inducing colonic adenomas via its tumour-promoting effects [38]. Another potential indirect mechanism for this effect is the increase in stress hormones, such as cortisol, affecting the tumour environment [39]. The findings from observational studies in humans included in the present meta-analysis, however, are inconsistent. A case–control study nested in four prospective studies (the Nurses’ Health Study, the Women’s Health Study, the Health Professional Follow-Up Study, and the Physicians’ Health Study II), which had 732 CRC cases, did not report any association between estradiol concentrations and CRC risk in men or women [2]. However, the same study found an inverse association of testosterone and SHBG concentrations with CRC in men but not in women [2]. In another case–control study nested in the female WHI-CT, no associations were observed between estradiol concentrations and CRC, whereas higher SHBG concentrations were associated with a higher CRC incidence [25]. In contrast, endogenous estradiol concentrations were positively associated with risk of CRC in the female WHI-OS [18]. Other studies with fewer CRC cases, ranging from 48 to 269, namely the Copenhagen City Heart Study [22], the NYUWHS cohort study [19], the JPHC Study Cohort II [20], and the Busselton Health Study [24] did not report any significant associations between endogenous sex hormone concentrations and CRC risk.

Possible explanations for these discrepancies include differences in the hormone assessment methods, variation in the number of cases and adjustments for confounders. Most included studies adjusted for several factors that may confound the sex hormone and CRC association such as adiposity [40], physical activity [41], smoking, and alcohol intake [42]. However, physical activity, smoking and alcohol were not included as confounders in the NYUWHS [19], and physical activity was not included in the Copenhagen City Heart Study [22]. Variation also existed as a result of the different hormone measurement methods used such as RIA, LC–MS/MS, and electrochemiluminescence [43]. For example, the median SHBG concentrations varied substantially between the studies, namely from 42.4 [25] to 221.2 nmol/L (converted from 63.8 ng/ml) [20] in the female controls. This could partially explain the large heterogeneity observed among the studies examining the relationship between SHBG and CRC in post-menopausal women (I^2^ = 76%). In the NYUWHS, there was a large amount of missing data (28.6%) and a high degree of laboratory error in the estradiol measurements (intra-batch coefficient of variation = 33.5%) [19], which might have biased the measurements and the reported associations. Standardization of high-quality sex hormone assays is needed for the validity of epidemiological studies and to draw definitive quantitative conclusions [44].

The strengths of this systematic review and meta-analysis include: (1) the prospective design and long follow-up period of the studies; (2) the inclusion of studies with populations largely free from exogenous administration of sex steroid hormones close to the time of assessment; (3) the inclusion of high-quality studies, as assessed with the “Newcastle–Ottawa Scale”.

Several limitations should also be noted. First, due to the observational nature of the studies included, residual confounding cannot be ruled out [45]. Second, the studies we meta-analysed relied on the measurement of sex hormone concentrations only once at baseline, and it is possible that this measurement may not reflect exposure levels across time. However, a previous analysis of postmenopausal women reported that the within-subject correlation coefficients for estradiol, testosterone and SHBG over a 2–3-year period were 0.64, 0.88 and 0.92 respectively [46], indicating that single measurements may be reasonably reliable of longer-term exposures. Third, the current available studies were relatively small and they did not provide results by important potential colorectal cancer subtypes (colon vs. rectal) and subgroups (age, adiposity). Only the study by Murphy et al., reported on separate analyses by colon vs. rectal cancer, but statistically significant differences were not observed by disease subtype for estradiol and SHBG concentrations [25]. Furthermore, it was not possible to investigate associations with young onset colorectal cancer, and future studies are warranted to investigate this disease, the incidence of which is increasing lately [47, 48].

In conclusion, the results of this systematic review and meta-analysis of prospective studies on the associations between endogenous sex hormone concentrations and CRC risk indicate that circulating concentrations of estradiol, testosterone and SHBG do not appear to be associated with risk of CRC in men and post-menopausal women. The observed lack of consistency in findings among the single studies suggests a need for more research in this area, with a particular focus on large prospective studies and reliable sex hormone assessments.

## Supplementary Information

Below is the link to the electronic supplementary material.

**Additional file 1**: **Table S1**. PubMed and Scopus search strategies. **Table S2**. Quality assessment of the prospective cohort studies, based on the NEWCASTLE-OTTAWA scale^a^. **Table S3**. Quality assessment of the nested case-control studies, based on the NEWCASTLE-OTTAWA scale^a^. **Figure S1**. Top versus bottom meta-analysed of the endogenous sex hormone concentrations. (PDF 71 KB)

## Data Availability

Not applicable.
