# Supplementary material for: Endogenous sex steroid hormones and colorectal cancer risk: a systematic review and meta-analysis
Source: Discov Oncol. 2021 Mar 15;12:8. doi: 10.1007/s12672-021-00402-z (PMC8777537; doi:10.1007/s12672-021-00402-z)
Supplement: Supplementary file 1 — Additional file 1: Table S1. PubMed and Scopus search strategies. Table S2. Quality assessment of the prospective cohort studies, based on the NEWCASTLE-OTTAWA scalea. Table S3. Quality assessment of the nested case-control studies, based on the NEWCASTLE-OTTAWA scalea. Figure S1. Top versus bottom meta-analysed of the endogenous sex hormone concentrations. (PDF 71 KB) [file 12672_2021_402_MOESM1_ESM.pdf]

Supplemental Table 1. PubMed and Scopus search strategies.

**PubMed**	
	Keywords for Sex hormones:
# 1	(gonadal steroid hormones) OR (sex hormones) OR estrogens OR androgens OR (sex hormone binding globulin) OR shbg OR estradiol OR oestradiol OR testosterone
	MeSH terms for Sex hormones:
# 2	(Gonadal Steroid Hormones) OR Estrogens OR Androgens OR (Sex Hormone-Binding Globulin) OR Estradiol OR Testosterone
	Keywords for colorectum:
# 3	(Colon OR Rectal OR Rectum OR Intestin* OR Colorectal OR CRC OR bowel OR gut)
	Keywords for cancer
# 4	(cancer OR neoplasm OR neoplasms OR carcinoma OR tumour OR tumor OR malignan*)
	MeSH terms for colorectal cancer:
# 5	(Colorectal neoplasms) OR (Colonic Neoplasms) OR (Rectal Neoplasms) OR (Sigmoid Neoplasms)
**(#1 OR #2) AND ((#3 AND #4) OR #5) AND english[Language] NOT review[PT] NOT animal[Filter]**	
**Scopus**	
	TITLE-ABS((gonadal steroid hormones) OR (sex hormones) OR estrogens OR androgens OR (sex hormone binding globulin) OR shbg OR estradiol OR oestradiol OR testosterone) **AND** TITLE-ABS(Colon OR Rectal OR Rectum OR Intestine OR intestinal OR Colorectal OR CRC OR bowel OR gut) **AND** TITLE-ABS(cancer OR neoplasm OR neoplasms OR carcinoma OR tumour OR tumor OR malignancy OR malignant) **AND** DOCTYPE(ar) **AND** LANGUAGE(english)

Supplemental Table 2. Quality assessment of the prospective cohort studies, based on the NEWCASTLE-OTTAWA scale.[a]

Study	Representativeness of the Exposed Cohort	Selection of the Non-Exposed Cohort	Ascertainment of Exposure	Demonstration That Outcome of Interest Was Not Present at Start of Study	Comparability of Cohorts on the Basis of the Design or Analysis	Assessment of Outcome	Was Follow-Up Long Enough for Outcomes to Occur	Adequacy of Follow Up of Cohorts
Orsted, D. D., 2014	*	*	*	*		*	*	*
Chan, Y. X., 2018	*	*	*	*	**	*	*	*
Chan, Y. X., 2017	*	*	*	*	**	*	*	*
Gunter, M. J., 2008	*	*	*	*	**	*	*	*

[a]A study can be awarded a maximum of one star for each numbered item within the Selection and Outcome categories. A maximum of two stars can be given for Comparability

Supplemental Table 3. Quality assessment of the nested case-control studies, based on the NEWCASTLE-OTTAWA scale. [a]

Study	Is the case definition adequate?	Representativeness of the cases	Selection of Controls	Definition of Controls	Comparability of cases and controls on the basis of the design or analysis	Ascertainment of exposure	Same method of ascertainment for cases and controls	Non-Response rate
Clendenen, T. V., 2009	*	*	*	*	*	*	*	*
Murphy, N., 2015	*	*	*	*	**	*	*	*
Mori, N., 2019	*	*	*	*	**	*	*	*
Lin, J. H., 2013	*		*	*	**	*	*	*

[a]A study can be awarded a maximum of one star for each numbered item within the Selection and Exposure categories. A maximum of two stars can be given for Comparability

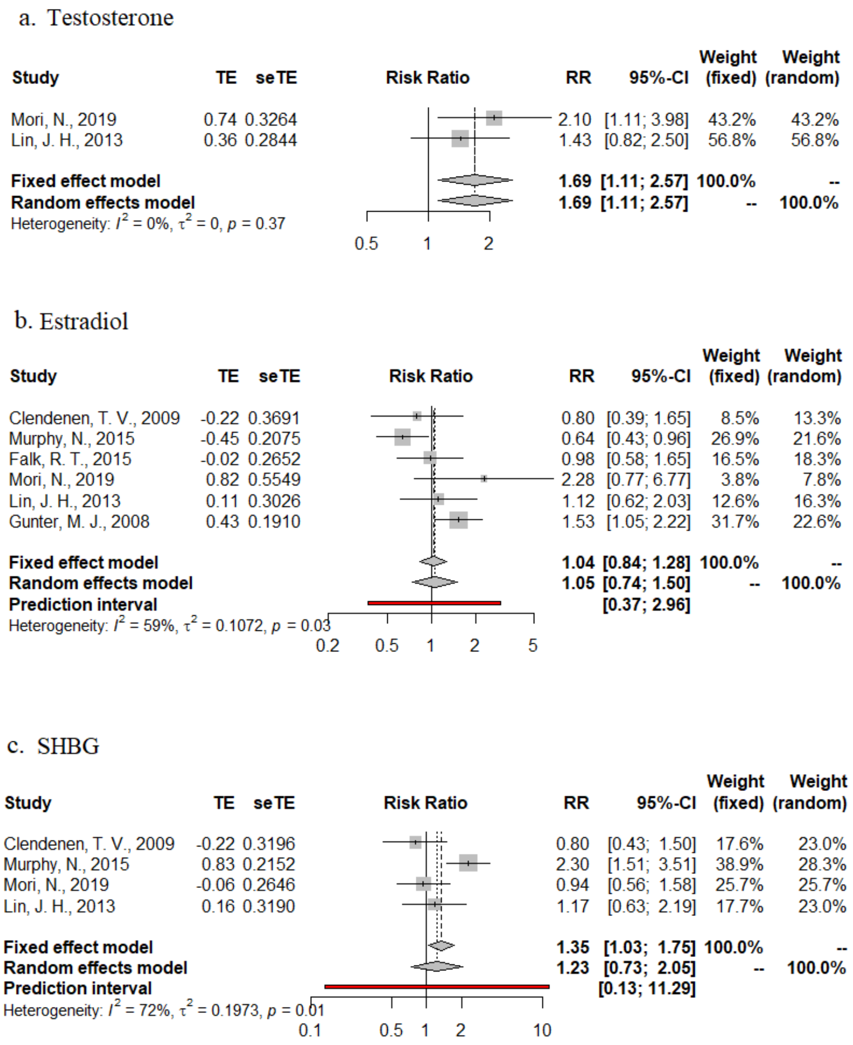

Supplementary Figure 1. Top versus bottom meta-analysed of the endogenous sex hormone concentrations.
